# Effect of Microcystins on Proto- and Metazooplankton Is More Evident in Artificial Than in Natural Waterbodies

**DOI:** 10.1007/s00248-017-1058-z

**Published:** 2017-09-02

**Authors:** J. Kosiba, W. Krztoń, E. Wilk-Woźniak

**Affiliations:** Department of Freshwater Biology, Institute of Nature Conservation, Polish Academy of Sciences, Al. Adama Mickiewicza 33, 31-120 Krakow, Poland

**Keywords:** Plankton, Oxbow lakes, Cyanobacterial blooms

## Abstract

The increasing proliferation of cyanobacterial blooms prolongs the impact of cyanobacteria on aquatic fauna, potentially altering trophic relationships. We hypothesized that any effect of dissolved microcystins (toxins produced by cyanobacteria) on plankton assemblages would be more evident in artificial reservoirs and ponds than in natural ones. The concentrations of dissolved microcystins in the waters we studied ranged widely from 0.07 to 0.81 μg/L. We showed that the artificial ponds were subjected to more frequent and longer-lasting harmful algal blooms. The plankton occurring in them were exposed to significantly higher concentrations of dissolved microcystins than those in natural oxbow lakes. Using a general linear model (GLM) regression, our study identified a significant relationship between dissolved microcystins and both the density and biomass of particular zooplankton groups (ciliates, rotifers, cladocerans, copepods). The density, biomass, and richness of the animal plankton were significantly lower in the artificial ponds than in the natural oxbow lakes. The impact of microcystins and the length of time that they remained in the water caused structural homogenization of the plankton.

**Electronic supplementary material:**

The online version of this article (10.1007/s00248-017-1058-z) contains supplementary material, which is available to authorized users.

## Introduction

Growing en masse in water, cyanobacteria create a phenomenon known as cyanobacterial blooms. Local and global warming and increasing anthropogenic eutrophication and pollution of water have led to the proliferation of harmful algal blooms (HABs) that show accelerated and prolonged activity [1]. Because “cyanoHABs” are toxic, cause hypoxia, decrease biodiversity, and disrupt food webs [2, 3], they present a serious threat to water ecosystems.

The most threatened ecosystems are those in small, shallow reservoirs, ponds, and oxbow lakes, which are biodiversity hotspots, serve as water migration corridors, diversify the landscape, and provide habitats for many rare and valuable species [4–6]. Because they are naturally eutrophic, these types of waterbodies naturally host cyanobacterial blooms, but the increasing proliferation of such blooms adds a new factor: it prolongs the impact of cyanobacteria on aquatic fauna, potentially altering trophic relationships, damaging these exceptionally important ecosystems, and compromising their ecosystem services.

Cyanobacteria change trophic interactions through several mechanisms. First, they are a poor food source due to their large size, low digestibility [7] and lack of long-chain polyunsaturated fatty acids (PUFAs) [8]. Second, they produce toxins. The most common of the several types of cyanotoxins are microcystins. Microcystins are produced by and retained in cyanobacterial cells during the growth and stationary phases of blooms [9]. When the blooms decay and their cells deteriorate, metabolites are released, raising the concentration of toxins in the water. The presence of microcystins is reported in 50 to 90% of samples taken during bloom events [10]. Toxins released in the water can remain there for up to 3 weeks [11], causing harm even after the cyanobacteria are gone. More than 100 microcystin analogues are known [12]. The analogues differ in toxicity; microcystin-LR (MC-LR) has been found to be the most toxic one, followed by microcystin-YR (MC-YR) and microcystin-RR (MC-RR) [13]. It is well known that microcystins harm humans and other mammals by altering cell metabolism and triggering a cascade of events that leads to cell necrosis or apoptosis [14]. Such effects do not require direct contact with cyanobacteria cells and occur even if the toxins cannot readily diffuse across the plasma membrane. There is evidence that hydrophobic toxins (e.g., MC-YR) can affect membranes that have packing defects [15]. Some hydrophobic microcystins can, by pinocytosis, penetrate the cell along with other material associated with the plasma membrane [16].

Dissolved cyanobacterial toxins released during bloom decay have negative effects on feeding and on the growth of fish larvae [17]. Cyanotoxins may be transferred to higher trophic levels through primary consumers such as protozooplankton [18] and metazooplankton [19]. Relatively little is known about the response of plankton to toxins, especially to dissolved toxins. It is difficult to draw conclusions about the processes and relationships that operate during CyanoHAB events, and effects measured in the laboratory may not always mirror the natural processes that occur in the field [20].

Finally, cyanotoxins may harm humans following chronic exposure to low concentrations of microcystins via consumption of contaminated water and food (e.g., agricultural products, fish, prawns, mollusks), dermal exposure, and inhalation [14].

Some species feed on cyanobacteria and are exposed to the toxins present in cyanobacterial cells. Many more species are exposed to cyanotoxins dissolved in the water. It is ever more important to understand how the presence of dissolved microcystins affects the structure and trophic network of plankton communities. Some field and laboratory studies have shown that toxins dissolved in the water affect the protozooplankton and metazooplankton living there [21–23].

Protozooplankton and metazooplankton organisms are basic and critical parts of the food web in aquatic ecosystems, able to transfer carbon to higher levels [24]. We studied the effect of dissolved microcystins on the shape of protozooplankton and metazooplankton assemblages in small waterbodies. With increasing anthropopression, we will see further proliferation of CyanoHABs. We need to know exactly how plankton assemblages will be affected by those blooms. For this study, we postulated that the effect of dissolved microcystins on plankton assemblages would be more pronounced in artificial waterbodies than in natural ones.

## Material and Methods

### Study Area and Materials

**Fig. 1:**
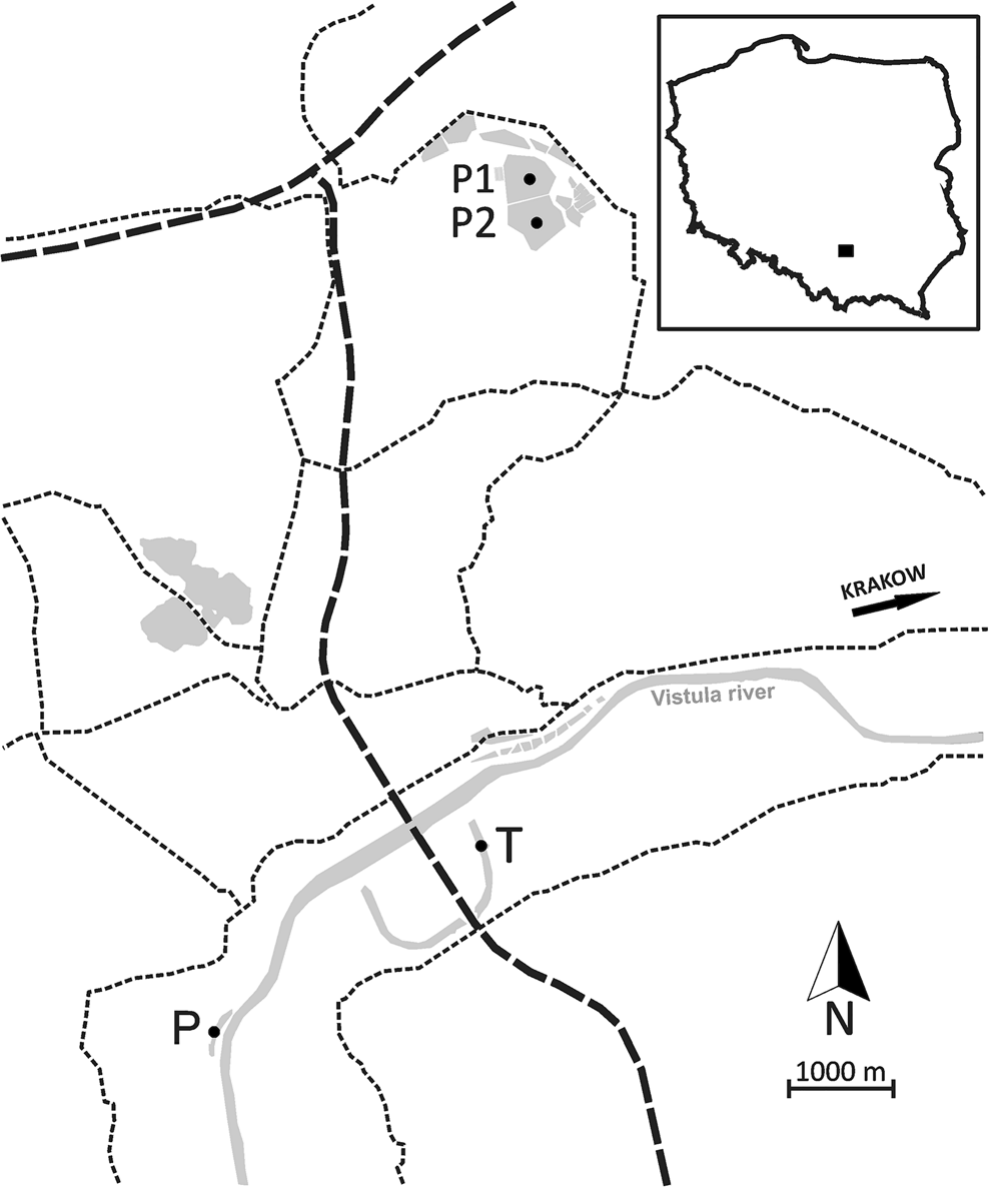
Locations of the studied waterbodies

This study used samples from four waterbodies in which cyanobacterial blooms occur: two natural oxbow lakes (Piekary, P; Tyniec, T) formed by the Vistula River and two artificial ponds (Podkamycze 1, P1; Podkamycze 2, P2) (Fig. 1). All the studied waterbodies are relatively small, covering 1.56–17.28 ha and ranging in maximum depth from 2.5 and 4.0 m. They all are classified as eutrophic [25] and are near each other, so their weather conditions are very similar.

### Sampling Procedure

Samples were collected from the central point of each waterbody between May and October 2014. Sampling was done each month before cyanobacterial blooms formed and then each week during bloom events. In total, 64 sample sets were collected for biological analyses (cyanobacteria, ciliates, metazooplankton) and to determine the concentration of microcystins in the water. Because the studied oxbow lakes are shallow and polymictic, they were not stratified into epilimnion, metalimnion and hypolimnion.

Although, the Ruttner sampler is not a perfect device for quantifying zooplankton abundance [26, 27], but it is broadly use in ecological studies [28]; therefore, we decided to use it. Samples were taken at 1 m depth using a 5-L Ruttner sampler and were concentrated from 10 L with plankton nets (mesh sizes 10 μm for cyanobacteria and ciliates; 50 μm for metazooplankton). Immediately after collection, the samples were fixed for quantitative analyses (with Lugol’s solution for algae and ciliates; with 4% formaldehyde for metazooplankton). Additional fresh (not fixed) samples were concentrated as described above, and the live material was taken for species composition analysis. See [24] for the keys used for taxonomic identification of cyanobacteria. The living ciliates were identified in 1 mL chambers with glass covers, according to [28] and [29], and their density was averaged from three counts. Total biomass of ciliates was calculated according to [30–33].

Metazooplankton samples were analyzed in 0.5 mL chambers, and their density was calculated as means of five counts. The keys we used for identification of animal species are listed in [23]. Dry weight was calculated by a regression equation defining the body length and weight of each species (see [23] for references). Because the phytoplankton and ciliates were calculated as fresh biomass, zooplankton dry mass was recalculated according to the index proposed by [34]. All microscopy of phytoplankton, ciliates, and metazooplankton employed a Nikon H550L light microscope at × 40–× 1000.

### Toxin Analysis

Microcystin concentrations (analogues: MC-LR, MC-RR, MC-YR) were determined by high-performance liquid chromatography (HPLC) using an Agilent 1100 apparatus with a diode matrix (DAD) in the Central Laboratory of the Municipal Water and Sewage Company in Krakow, Poland [35].

### Statistical Analysis

The Mann-Whitney *U* test was used to ascertain the statistical significance of differences between the artificial ponds and natural oxbow lakes. The factors analyzed included the microcystin concentrations and the population parameters for the protozooplankton (*Ciliata*), metazooplankton, and particular groups of metazooplankton (*Cladocera*, *Copepoda*, *Rotifera*). Canonical correspondence analysis (CCA; constrained ordination) was applied to analyze the effect of type of waterbody on species composition; the same weight was given to each species in the analysis, regardless of the count of a given species in the samples.

We applied a set of generalized linear models (GLMs) to determine whether the density and biomass of the protozooplankton and metazooplankton depended on the dissolved microcystins, using Poisson error distributions for the density and biomass data from the different plankton groups. GLM residuals were graphically examined to test the model assumptions (residual distribution, independence, homoscedasticity). Finally, we used partial residual plots to visualize significant relationships between the density or biomass of the protozooplankton and metazooplankton and the dissolved microcystins. According to [36], both of the methods we used are good options for spatial modeling of species distributions.

All of our analyzed data were log-transformed. The statistical analyses employed Statistica 12 (descriptive statistics, Mann-Whitney *U* test), Past 3.10 (box plots), and Canoco 5.04 (CCA, GLM).

## Results

### Cyanobacterial Blooms and Microcystins

**Fig. 2:**
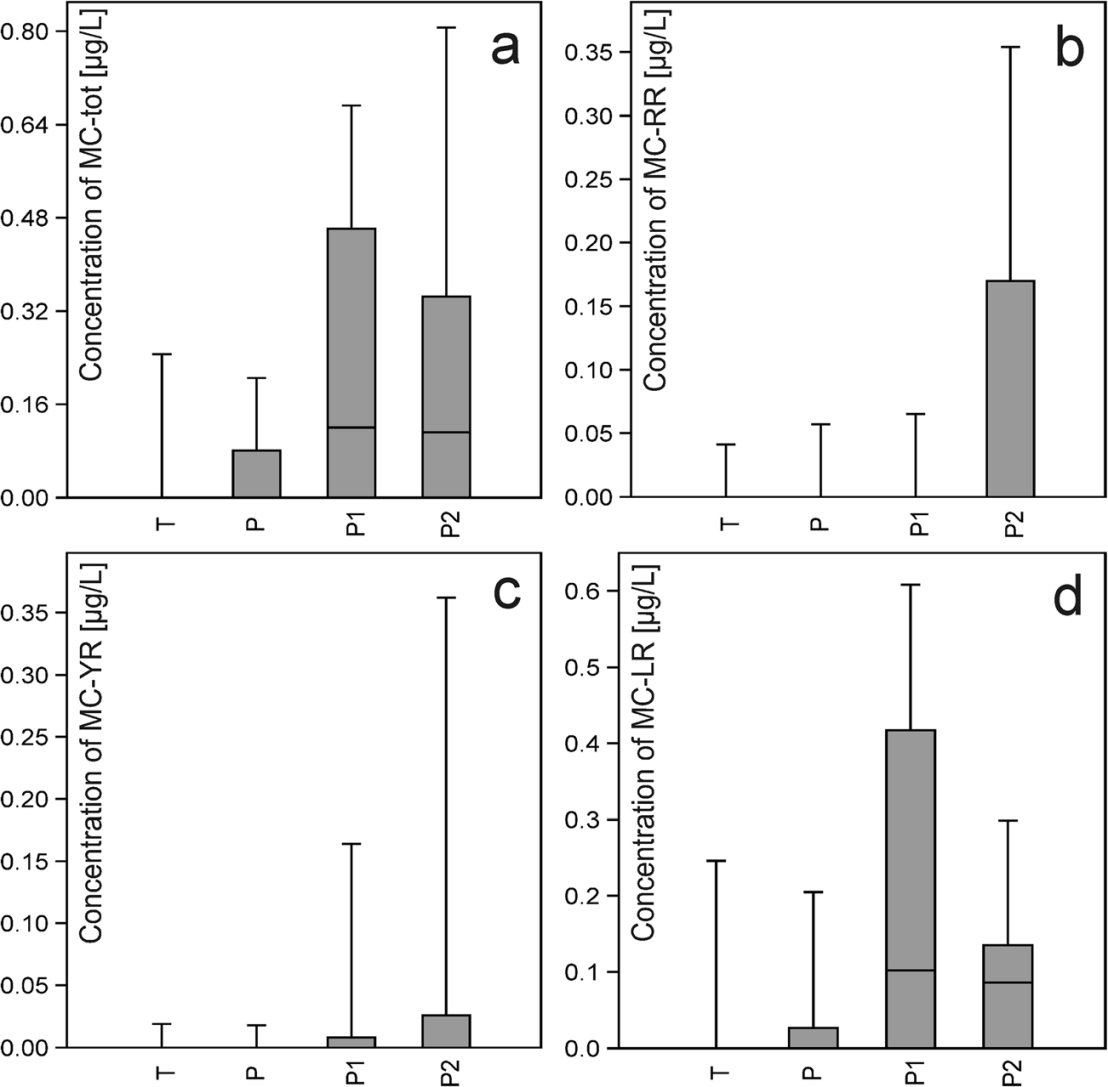
**a**–**d** Dissolved microcystin concentrations (μg/L) in the waterbodies. **a** MCtot. **b** MC-RR. **c** MC-YR. **d** MC-LR. Dark horizontal lines represent medians; boxes enclose 25th and 75th percentiles

Cyanobacterial blooms were observed in all four waterbodies. The blooms persisted for up to 3 months in the two oxbow lakes (P, T) and for up to 6 months in the two artificial ponds (P1, P2). Cyanobacterial toxins (microcystins) occurred in the water of all studied waterbodies but varied in concentration and duration (Table 1; Fig. 2).

**Table 1:** Basic information about the type of waterbody, cyanobacterial blooms, and microcystin concentrations

	Piekary	Tyniec	Podkamycze 1	Podkamycze 2
Geographical coordinates	50° 00′ 50.1″ N, 19° 47′ 35.7″ E	50° 01′ 47″ N, 19° 49′ 39.8″ E	50° 05′ 11″ N, 19° 50′ 01.6″ E	50° 04′ 59.6″ N, 19° 50′ 05.4″ E
Type of reservoir	Natural	Natural	Artificial	Artificial
Max depth (m)	4.0	3.0	3.0	2.5
Area (ha)	1.56	5.75	16.82	17.28
Trophic class	Eutrophic	Eutrophic	Eutrophic	Eutrophic
Period of bloom	From August to October	From August to October	From May to November	From May to November
Species created blooms	*Oscillatoria tenuis*, *Dolichospermum planctonicum*, *D. spiroides*, *Microcystis wesenbergii*	*Aphanocapsa* sp., *Microcystis aeruginosa*, *M. ichtyblabe*, *M. wesenbergii*, *Woronichiania naegeliana*, *Aphanizomenon* sp.	*Aphanizomenon flos-aque* with *M. aeruginosa*	*Aphanizomenon flos-aque* with *M. aeruginosa*
Presence of microcystins dissolved in water	All of October	Beginning of September and end of October	From end of June to August and from mid-September to end of October	From end of June to beginning of August and from mid-September to end of October
Concentration of toxins (MCtot)	Min.–max. = 0.00–0.21 μg/L; Avg. = 0.07 μg/L; SD = 0.09 μg/L	Min.–max. = 0.00–0.25 μg/L; Avg. = 0.03 μg/L; SD = 0.08 μg/L	Min.–max. = 0.00–0.67 μg/L; Avg. = 0.17 μg/L; SD = 0.21 μg/L	Min.–max. = 0.00–0.81 μg/L; Avg. = 0.19 μg/L; SD = 0.24 μg/L

*Avg.* average, *max.* maximum, *min.* minimum, *SD* standard deviation

The dissolved microcystin concentrations were highest in the artificial ponds (P1, P2) and varied the most in P2 (Fig. 2a); the concentrations were lower and more uniform in the natural oxbow lakes (P, T) (Fig. 2b–d). The microcystin forms differed in their patterns of occurrence: in the artificial ponds, the highest concentration of MC-LR was found in P1 and the highest concentration MC-RR and MC-YR in P2 (Fig. 2b–d).

The differences in dissolved microcystin concentrations between the natural oxbow lakes and the artificial ponds were statistically significant (for MCtot Mann-Whitney *U* test, *z* = − 3.00 and *p* < 0.000; for MC-LR Mann-Whitney *U* test, *z* = − 2.43 and *p* = 0.015).

### Zooplankton Structure

The zooplankton organisms were divided into protozooplankton (*Ciliata*) and metazooplankton (*Cladocera*, *Copepoda*, *Rotifera*). We recorded 15 *Ciliata* taxa and 54 metazooplankton taxa (see supplementary data). The average number of *Ciliata* taxa was lower than the average number of metazoan taxa, but Spearman rank correlations showed a positive relationship between the number of *Ciliata* taxa and the number of metazooplankton taxa (*r* = 0.46, *p* < 0.05).

**Fig. 3:**
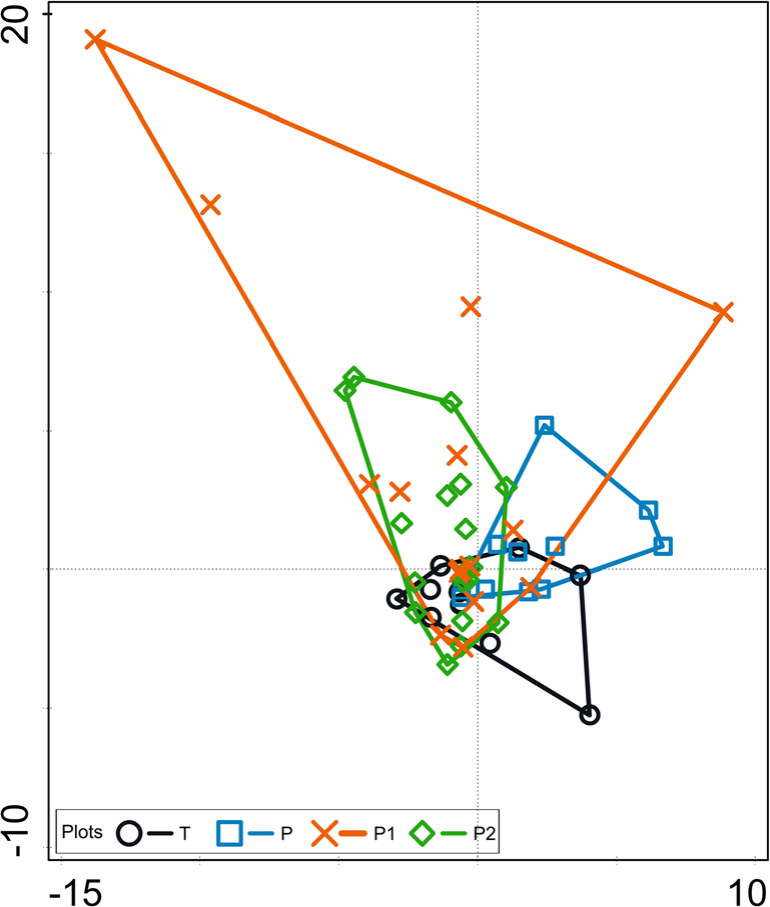
CCA plot diagram. Composition of *Ciliata* assemblages, samples, and waterbodies. The samples are grouped as follows: blue envelope—Piekary oxbow lake (natural reservoir); black envelope—Tyniec oxbow lake (natural reservoir); brown and green envelopes—Podkamycze 1 and 2 (artificial ponds). Total variation = 3.24; explanatory variables account for 4.0%. Eigenvalues for axis 1 = 0.067 and for axis 2 = 0.042. Permutation test results: on first axis pseudo-*F* = 1.2, *P* = 0.81; on all axes pseudo-*F* = 0.8, *P* = 0.836. Explained fitted variation (cumulative) for axis 1 = 51.94 and axis 2 = 84.33.

CCA partially differentiated the protozooplankton of the natural waterbodies (P, T) from that of the artificial ponds (P1, P2) along the first axis based on the species composition of the samples, but those results were not statistically significant (Fig. 3).

**Fig. 4:**
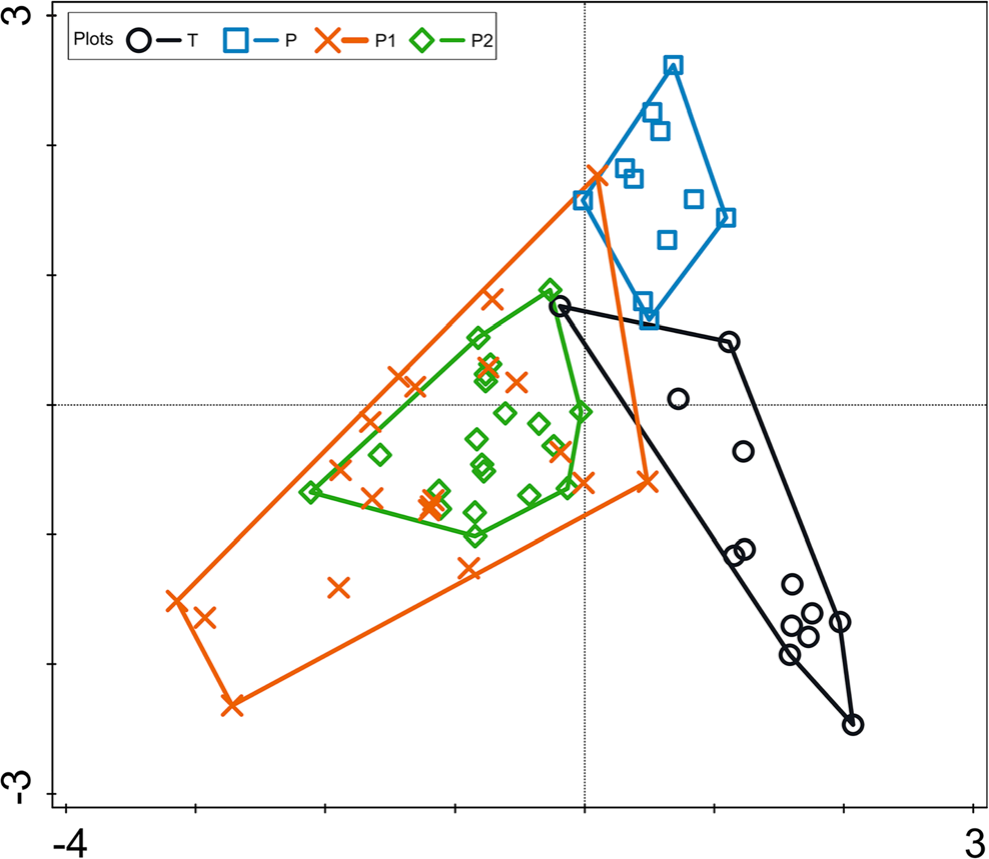
CCA plot diagram. Composition of metazooplankton assemblages, samples, and waterbodies. Samples are grouped as follows: blue envelope—Piekary oxbow lake (natural reservoir); black envelope—Tyniec oxbow lake (natural reservoir); brown and green envelopes—Podkamycze 1 and 2 (artificial ponds). Total variation = 2.74; explanatory variables account for 13.9%. Eigenvalues for axis 1 = 0.207 and for axis 2 = 0.097. Permutation test results: on first axis pseudo-*F* = 4.9, *P* = 0.002; on all axes pseudo-*F* = 3.2, *P* = 0.002. Explained fitted variation (cumulative) for axis 1 = 54.29 and axis 2 = 79.81

CCA of the metazooplankton showed differences in species composition between the natural (P, T) and artificial (P1, P2) waterbodies along the first axis based on the species composition of the samples. Those differences were statistically significant (Fig. 4).

### Zooplankton vs. Dissolved Microcystins

GLM showed statistically significant negative relationships between the biomass and the density of several zooplankton groups and the concentrations of MCtot and MC-LR (Tables 2 and 3), but not for MC-RR or MC-YR.

**Table 2:** GLM, biomass of protozooplankton, and particular groups of metazooplankton and microcystins (MCtot and MC-LR) dissolved in water

Response variable	Predictors	Fitted model deviance	Null deviance	Model AIC	Model test *F*	*p*	*B* intercept/MC tot or MC-LR	s.e. intercept/MC tot or MC-LR	*T* value intercept/MC tot or MC-LR
Total biomass of *Ciliata*	MCtot	320.77	348.89	409.54	28.1	< 0.000	1.20/− 3.17	0.09/0.71	12.33/− 4.41
Total biomass of *Ciliata*	MC-LR	305.27	330.70	389.57	25.4	< 0.000	1.26/− 4.27	0.09/1.03	13.03/− 4.14
Total biomass of metazooplankton	MCtot	227.74	260.30	429.18	32.6	< 0.000	2.29/− 1.59	0.05/0.30	41.48/− 5.21
Total biomass of metazooplankton	MC-LR	226.40	245.93	405.97	19.5	< 0.000	2.19/− 1.81	0.06/0.44	37.27/− 4.06
Biomass of *Copepod*a	MCtot	131.33	135.99	285.17	4.7	0.035	1.40/− 0.83	0.08/0.40	16.51/− 2.06
Biomass of *Copepoda*	MC-LR	125.63	132.64	265.16	7.0	0.010	1.44/− 1.52	0.08/0.61	16.88/− 2.46
Biomass of *Cladocera*	MCtot	315.85	361.06	434.71	45.2	< 0.000	1.51/− 3.66	0.08/0.67	17.99/− 5.44
Biomass of *Cladocera*	MC-LR	297.31	313.73	395.70	16.4	< 0.000	1.18/− 3.19	0.09/0.91	11.96/− 3.49

Only statistically significant relationships are show

**Table 3:** GLM, density of protozooplankton, and particular groups of metazooplankton and microcystins (MCtot and MC-LR) dissolved in water

Response variable	predictors	Fitted model deviance	Null deviance	Model AIC	Model test *F*	*p*	*B* intercept/MC tot or MC-LR	s.e. intercept/MC tot or MC-LR	*T* value intercept/MC tot or MC-LR
Total density of *Ciliata*	MCtot	17,002,628.19	19,021,611.61	1.7e+007	2.019e+006	< 0.000	12.03/− 4.41	0.0004/0.004	27,099.8/− 1103.35
Total density of *Ciliata*	MC-LR	16,044,830.96	17,942,766.49	1.605e+007	1.898e+006	< 0.000	12.09/− 6.15	0.0004/0.006	27,638.6/− 1050.42
Total density of Metazooplankton	MCtot	77,365.64	79,328.20	7.782e+004	1963	< 0.000	7.28/− 0.92	0.005/0.022	1616.07/− 42.12
Total density of Metazooplankton	MC-LR	75,349.29	76,393.39	7.577e+004	1044	< 0.000	7.29/− 0.93	0.005/0.030	1611.15/− 30.98
Density of *Copepoda*	MCtot	5339.32	5643.18	5704.77	303.9	< 0.000	5.34/− 0.96	0.012/0.058	451.08/− 16.54
Density of *Copepoda*	MC-LR	4988.59	5366.55	5321.14	378.0	< 0.000	5.38/− 1.57	0.012/0.087	450.06/− 18.08
Density of *Cladocera*	MCtot	3739.79	4301.09	4022.47	561.3	< 0.000	4.50/− 2.50	0.019/0.12	241.49/− 20.51
Density of *Cladocera*	MC-LR	3150.87	3381.86	3398.86	231.0	< 0.000	4.24/− 2.39	0.021/0.175	198.68/− 13.60
Density of *Rotifera*	MCtot	86,520.67	87,833.35	8.694e+004	1313	< 0.000	7.05/− 0.83	0.005/0.024	1397.82/− 34.62
Density of *Rotifera*	MC-LR	82,454.17	83,053.02	8.283e+004	598.9	< 0.000	7.08/− 0.764	0.005/0.0326	1408.89/− 23.64

Only statistically significant relationships are shown

### Population Parameters of Proto- and Metazooplankton Assemblages

**Fig. 5:**
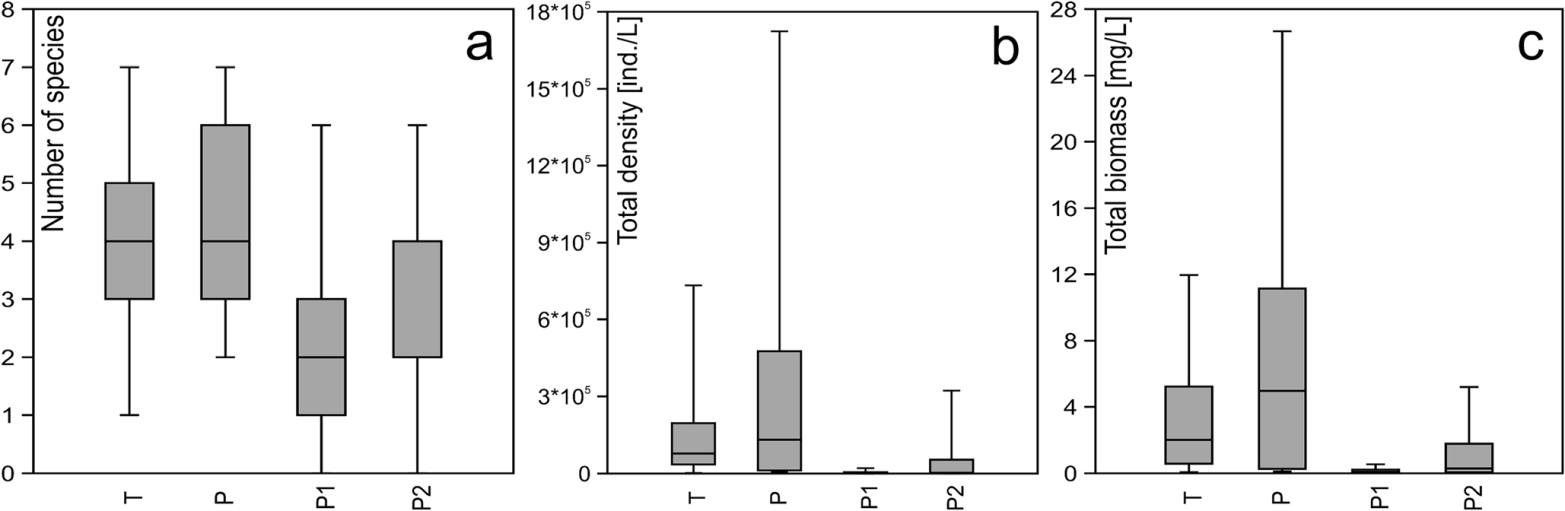
Box plots for **a** number of species, **b** total density, and **c** total biomass of *Ciliata* in particular waterbodies. Mann-Whitney *U* test showed statistically significant differences between the natural (P, T) and artificial waterbodies (P1, P2) for all parameters (number of species *z* = 4.215, *p* < 0.000; density *z* = 4.833, *p* < 0.000; biomass *z* = 4.472, *p* < 0.000). Dark horizontal lines represent medians; boxes enclose 25th and 75th percentiles; whiskers represent 5th and 95th percentiles

The richness, total density, and total biomass of *Ciliata* species in the natural oxbow lakes (P, T), having lower microcystin concentrations, were significantly higher than in the artificial ponds (P1, P2), having higher microcystin concentrations (Fig. 5a–c).

**Fig. 6:**
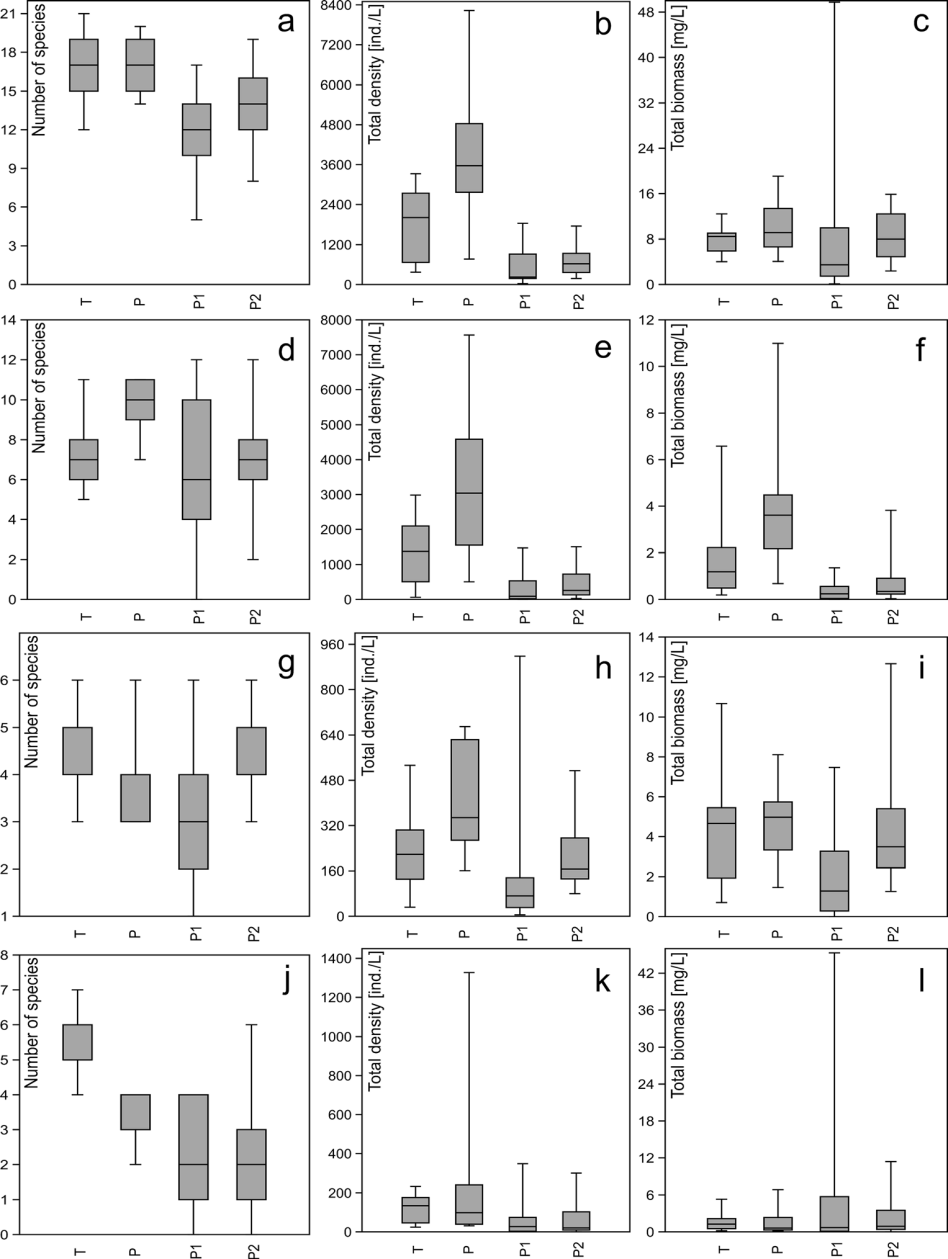
Box plots. **a** Total number of metazooplankton species (Mann-Whitney *U* test *z* = 5.001, *p* < 0.000). **b** Total density of metazooplankton (Mann-Whitney *U* test *z* = 5.235, *p* < 0.000). **c** Total biomass of metazooplankton (not statistically significant). **d** Total number of *Rotifera* species (Mann-Whitney *U z* = 2.039, *p* = 0.041). **e** Total density of *Rotifera* (Mann-Whitney *U* test *z* = 5.151, *p* < 0.000). **f** Total biomass of *Rotifera* (Mann-Whitney *U* test *z* = 4.937, *p* < 0.000). **g** Total number of *Copepoda* species (not statistically significant). **h** Total density of *Copepoda* (Mann-Whitney *U* test *z* = 3.314, *p* < 0.000). **i** Total biomass of *Copepoda* (Mann-Whitney *U* test *z* = 2.364, *p* = 0.018). **j** Total number of *Cladocera* species (Mann-Whitney *U* test *z* = 5.077, *p* < 0.000). **k** Total density of *Cladocera* (Mann-Whitney *U* test *z* = 3.842, *p* < 0.000). **l** Total biomass of *Cladocera* (not statistically significant). Dark horizontal lines represent medians; boxes enclose 25th and 75th percentiles; whiskers represent 5th and 95th percentiles

The richness and density of metazooplankton species were significantly higher in waterbodies that had shorter-duration cyanobacterial blooms and lower microcystin concentrations (Fig. 6a–c), but total metazooplankton biomass did not show such a correlation. The natural and artificial waterbodies differed significantly for biomass of *Rotifera* (Fig. 6d–f) and *Copepoda* (Fig. 6g–i), but surprisingly not for biomass of *Cladocera* (Fig. 6j–l).

## Discussion

Microcystins are a group of toxins often present in water, as they are produced by species that commonly occur there (e.g., species of the genera *Planktothrix*, *Microcystis*, *Aphanizomenon*, *Nostoc*, *Anabaena*) [37]. In the studied waterbodies, we found three microcystin analogues: MC-YR, MC-RR, and MC-LR. The first two occurred at small concentrations, and for them, we found no significant differences between the waterbodies nor any relationships with plankton parameters. Only dissolved MC-LR was associated with the parameters of the plankton, both protozooplankton (*Ciliata*) and metazooplankton. Differences in hydrophobicity can make microcystins differ in the way that they are taken up by animals. They may be ingested with food [38] or may bind to membranes and penetrate cells by pinocytosis [16]. The microcystins affected the plankton animals in different ways in the studied waterbodies. We showed that they were more harmful to these organisms in the artificial ponds than in the natural oxbow lakes. There were significant differences in dissolved MC-LR concentration between the natural and artificial waterbodies. MC-LR is known to be the most potent toxin [39]; we infer that the significantly higher and longer-persisting concentrations of that analogue in the artificial ponds shaped the structure of the ciliate and metazooplankton assemblages.

Species-specific adaptations in zooplankton have led to variation of the observed responses to cyanobacteria blooms [40] and cyanobacterial toxins. In the literature, information about the response of ciliates [21, 22, 41, 42], rotifers [43, 44], copepods [20, 45], and cladocerans [46, 47] to cyanotoxins is contradictory and unclear. Our GLM analyses showed significant negative correlations between the dissolved microcystins and both the density and the biomass of *Ciliata*. Other research indicates that cyanobacterial blooms generally affect communities of ciliates by lowering their diversity: only a few ciliate species were found to develop during the culminating stage of cyanobacterial blooms [48].

The richness, total biomass, and density of *Ciliata* species in particular samples were significantly lower in the two artificial ponds (P1, P2), where microcystins occurred at significantly higher concentrations and remained in the water longer than in the oxbow lakes (P, T). The composition of *Ciliata* assemblages in particular samples was more uniform in the ponds and assumed a more typical structure in the oxbow lakes (CCA). That uniformity or homogeneity of *Ciliata* assemblages in the artificial ponds reflects their longer exposure to dissolved cyanotoxins. The more typical structure of the assemblages found in the oxbow lakes reflects the operation of an ecosystem in which toxins are present at lower concentrations and for a shorter period.

The response of the metazoan assemblages was similar to that of the ciliate assemblages. GLM regression showed negative relationships between dissolved microcystins and both the density and the biomass of the metazooplankton. We found significantly fewer species and lower total density of metazooplankton in the ponds (P1, P2) than in the oxbow lakes (P, T), but surprisingly we did not find significant differences in total biomass.

Since metazooplankton organisms form a heterogeneous group consisting of various subgroups, we also analyzed data from particular groups. We found a significant relationship between microcystins and the density of *Rotifera* and a decrease in the number of species, total density, and total biomass of rotifers in the ponds, which had higher dissolved microcystin concentrations.

Copepod biomass was also negatively correlated with dissolved microcystin concentration. However, copepods are able to discriminate between toxic and nontoxic cyanobacteria [44], but they can assimilate toxins directly from the water or via ciliates [49, 50], and they may adsorb toxins and then transfer them to higher trophic levels [51]. Analyses of copepod biomass and density showed statistically significant differences between the ponds (P1, P2) and the oxbow lakes (P, T), in line with laboratory studies [45] which showed that an elevated concentration of microcystins reduced the survival of *Eurytemora affinis.*

The relationship between toxins and *Cladocera* is even more complicated. It has been demonstrated that *Daphnia* species may adapt to the presence of toxins [47]. Small cladocerans such as *Bosmina* may not be sensitive to the effects of microcystins. *Bosmina* and *Daphnia* are species that ingest toxic cyanobacteria, leading to microcystin accumulation [52, 53] and transferring them to higher trophic levels [54]. In our study, *Cladocera* showed significant negative correlations with microcystins, mainly MC-LR. There were significant differences in the total density but not the biomass of *Cladocera* between the artificial and natural waterbodies: the oxbow lakes showed higher density of *Cladocera* species but their biomass was higher in the ponds. This suggests that the large cladocerans (*Daphnia*) in our waterbodies were adapted to higher concentrations of those toxins.

## Conclusion

We demonstrated that in waterbodies with higher and longer-persisting microcystin concentrations, various parameters (density, biomass, richness) of the zooplankton population decreased, and the structure of the species assemblages tended toward uniformity. The studied artificial ponds were more exposed to harmful cyanobacterial blooms, and for a longer period, than the natural oxbow lakes. The general problem can be expressed in this way: increasing artificiality of the aquatic environment (transformation, destruction, creation of new waterbodies) + eutrophication + global warming = increased proliferation of toxic cyanobacterial blooms + homogenization of plankton species structure.

## Electronic Supplementary Material

ESM 1 (DOC 105 kb)
