# Supplementary material for: Effect of Microcystins on Proto- and Metazooplankton Is More Evident in Artificial Than in Natural Waterbodies
Source: Microb Ecol. 2017 Sep 2;75(2):293–302. doi: 10.1007/s00248-017-1058-z (PMC5742606; doi:10.1007/s00248-017-1058-z)
Supplement: Supplementary file 1 — (DOC 105 kb) [file 248_2017_1058_MOESM1_ESM.doc]

Supplementary data.

List of proto- and metazooplankton species found in studied water bodies

| **Group,** *species* | **PIEKARY** | **TYNIEC** | **PODKAMYCZE 1** | **PODKAMYCZE 2** |
| --- | --- | --- | --- | --- |
| **Protozooplankton** |  |  |  |  |
| *Aspidisca* sp. |  | + |  | + |
| *Codonella cratera*  (Leidy, 1887) | + | + | + | + |
| *Coleps hirtus* (Ehrenberg, 1831) | + | + | + | + |
| *Coleps spetai* (Foissner, 1984) | + | + | + | + |
| *Epistylis* sp. | + | + | + | + |
| Non-identified | + | + |  |  |
| *Paramecium* sp. |  |  | + |  |
| small scuticociliata |  |  | + | + |
| *Stentor* sp. | + | + | + | + |
| *Strobilidium* sp. | + | + | + |  |
| *Strombidium* sp. |  |  | + | + |
| *Tintinidium* sp. | + | + | + | + |
| *Vorticella campanula* (Ehrenberg, 1831) |  | + |  |  |
| *Vorticella convalaria* complex |  |  |  | + |
| *Vorticella* sp. |  | + | + | + |
| **Metazooplankton** |  |  |  |  |
| **Rotifera** |  |  |  |  |
| *Asplanchna priodonta*  (Gosse, 1850) | + | + | + | + |
| *Brachionus angularis*  (Gosse, 1851) | + | + | + | + |
| *Brachinus calyciforus*  (Palas, 1766) | + | + | + | + |
| *Brachionus diversicornis* (Daday, 1883) | + | + | + | + |
| *Brachionus quadridentatus* (Hermann, 1783) |  |  | + | + |
| *Brachionus rubens* (Ehrenberg, 1838) |  |  | + | + |
| *Brachionus urceolaris*  (Műller, 1773) | + |  | + |  |
| *Filinia longiseta* (Ehrenberg, 1834) | + | + | + | + |
| *Gastropus minor* (Rousselet, 1892) | + |  | + |  |
| *Kellicotia longispina* (Kellicott, 1879) | + | + | + | + |
| *Keratella cochlearis* (Gosse, 1851) | + | + | + | + |
| *Keratella quadrata* (Műller, 1786) | + | + | + | + |
| *Keratella tecta* (Gosse, 1851) | + | + | + | + |
| *Lecane* sp. |  |  | + | + |
| *Polyarthra longiremis*  (Carlin. 1943) |  |  | + | + |
| *Polyarthra major* (Burckhardt, 1900) | + | + | + | + |
| *Polyarthra minor* (Voigt, 1904) | + |  | + | + |
| *Polyarthra remata* (Skorikov, 1896) | + | + | + | + |
| *Polyarthra vulgaris* (Carlin, 1943) | + | + | + | + |
| *Pompholyx sulcata* (Hudson, 1885) | + | + | + | + |
| *Synchaeta pectinata* (Ehrenberg, 1832) |  |  | + |  |
| *Trichocerca capucina* (Wierzejski & Zacharias, 1893) | + | + | + | + |
| *Trichocerca similis* (Wierzejski, 1893) | + | + |  | + |
| **Copepoda** |  |  |  |  |
| *Acanthocyclops robustus*  (G.O. Sars, 1863) |  |  |  | + |
| *Acanthocyclops trajani* (Mirabdullayev & Defaye, 2002) |  |  |  | + |
| *Acantocyclops venustus*  (Norman & Scott, 1906) | + | + | + | + |
| Copepodid | + | + | + | + |
| *Cyclops abyssorum* (G.O. Sars, 1863) |  | + |  |  |
| *Cyclops strenuus* (Fischer, 1851) |  | + | + | + |
| *Cyclops vicinus* (Uljanin, 1875) | + | + | + | + |
| *Eudiaptomus gracilis*  G.O. Sars, 1863) | + | + | + | + |
| *Eurytemora affinis* (Poppe, 1880) | + |  |  |  |
| *Mesocyclops leuckatrii*  (Clauss, 1857) |  | + |  |  |
| *Metacyclops gracilis* (Lilljeborg, 1853) |  | + |  |  |
| Nauplius | + | + | + | + |
| *Thermocyclops crassus*  (Fischer, 1853) | + | + | + | + |
| *Thermocyclops dybowskii*  (Landé, 1890) |  |  | + |  |
| *Thermocyclops oithonoides*  (G.O. Sars, 1863) |  |  |  | + |
| **Cladocera** |  |  |  |  |
| *Alona affinis* (Leydig, 1860) |  |  | + |  |
| *Bosmina longirostris*  (O.F. Műller, 1785) | + | + | + | + |
| *Chydorus latus* (G.O. Sars, 1862) |  |  | + | + |
| *Chydorus sphaericus*  (O.F. Műller, 1786) | + | + | + |  |
| *Daphnia ambigua* (Scourfield, 1947) |  | + |  |  |
| *Daphnia cristata* (G.O. Sars, 1861) | + |  | + |  |
| *Daphnia cucullata* (G.O. Sars, 1862) | + | + | + | + |
| *Daphnia galeata* (G.O. Sars, 1864) |  | + | + | + |
| *Daphnia longispina* (O.F. Műller, 1776) |  | + | + | + |
| *Daphnia magna* (Straus, 1820) |  |  | + |  |
| *Diaphanosoma brachyurum*  (Liévin, 1848) | + | + | + |  |
| *Eubosmina coregoni* (Baird, 1857) |  | + |  | + |
| *Eubosmina gibera* (Schoedler, 1863) | + | + |  |  |
| *Eubosmina longispina*  (Leydig, 1860) | + | + | + | + |
| *Eurycercus lamellatus*  (O.F. Műller, 1776) |  |  | + |  |
| *Leptodora kindtii* (Focke, 1844) | + | + |  | + |
| *Moina micrura* (Kurz, 1875) | + | + |  |  |
| *Phreatalona protzi*  (Hartwig, 1900) comb. nov. =*Alona protzi* Hartwig, 1900 |  |  |  | + |
